# How does the metabolism of tumour cells differ from that of normal cells

**DOI:** 10.1042/BSR20130066

**Published:** 2013-11-15

**Authors:** Nívea Dias Amoêdo, Juan Perez Valencia, Mariana Figueiredo Rodrigues, Antonio Galina, Franklin David Rumjanek

**Affiliations:** *Instituto de Bioquímica Médica, Centro de Ciências da Saúde, Universidade Federal do Rio de Janeiro, Cidade Universitária, Av, Carlos Chagas Filho 373, Ilha do Fundão CEP 21941-590, Rio de Janeiro, Brazil

**Keywords:** energy, glutamine, glycolysis, hypoxia, lipids, mitochondria, 3BP, 3-bromopyruvate, ETS, electron transport system, GA, glutaminases, GSH, reduced glutathione, HIF, hypoxia-inducible factor, HK-II, hexokinase II, IDH, isocitrate dehydrogenase, MCT 1, monocarboxylate transporter 1, MPTP, mitochondrial permeability transition pore, PyK M2, pyruvate kinase isoform M2, ROS, reactive oxygen species, SDH, succinate desidrogenase, SERCA, sarcoplasmic/endoplasmic reticulum Ca^2+^-ATPase, TCA, tricaboxylic acid, TRAP-1, TNF (tumour necrosis factor) receptor-associated protein 1

## Abstract

Tumour cells thrive in environments that would be hostile to their normal cell counterparts. Survival depends on the selection of cell lines that harbour modifications of both, gene regulation that shifts the balance between the cell cycle and apoptosis and those that involve the plasticity of the metabolic machinery. With regards to metabolism, the selected phenotypes usually display enhanced anaerobic glycolysis even in the presence of oxygen, the so-called Warburg effect, and anabolic pathways that provide precursors for the synthesis of lipids, proteins and DNA. The review will discuss the original ideas of Otto Warburg and how they initially led to the notion that mitochondria of tumour cells were dysfunctional. Data will be presented to show that not only the organelles are viable and respiring, but that they are key players in tumorigenesis and metastasis. Likewise, interconnecting pathways that stand out in the tumour phenotype and that require intact mitochondria such as glutaminolysis will be addressed. Furthermore, comments will be made as to how the peculiarities of the biochemistry of tumour cells renders them amenable to new forms of treatment by highlighting possible targets for inhibitors. In this respect, a case study describing the effect of a metabolite analogue, the alkylating agent 3BP (3-bromopyruvate), on glycolytic enzyme targets will be presented.

Tumour cells, like other cells, are entirely dependent on an adequate supply of energy in order to support cellular events such as proliferation, migration and invasion as occurs with metastatic cells. From the biochemical point of view, proliferation alone encompasses several anabolic reactions, all costly in terms of energy and whose end products are the cell's raw materials, proteins, nucleic acids and lipids. With regards to bioenergetics it is well established that tumour cells not only survive, but thrive as a result of selection of metabolic pathways that can generate enough ATP and other metabolites even when underfed or subjected to hypoxia, conditions that that would certainly be harmful to most untransformed cells. Within the context of bioenergetics tumour cells have in fact become viable through the selection of pathways derived from a large repertoire of reactions that normally affords metabolic plasticity to normal cells. As a result, some patterns have emerged.

One of the most frequent and apparent peculiarities observed, at least in a great variety of solid tumours, is the prevalence of aerobic glycolysis over oxidative phosphorylation. Cells displaying this phenotype, also referred to as the Warburg effect, or the loss of the Pasteur effect, carry out aerobic glycolysis even in the presence of an ample supply of oxygen. In the clinical scenario aerobic glycolysis has been recognized as a typical feature of tumours, so much so that it actually forms the basis of the widely used diagnostic procedure of positron emission tomography that measures the uptake of fluorodeoxyglucose by tumour cells. However, it is known today that aerobic glycolysis *per se* cannot be generalized as the only or main source of energy for all types of cancer. Also it must be borne in mind that aerobic glycolysis is not exclusive to tumour cells. Lactate metabolism is the pathway of choice for some normal tissues such as the myocardium and brain, whose astrocytes are essentially glycolytic in spite of available oxygen.

Apart from aerobic glycolysis, tumour cells are notoriously dependent on glutamine for their survival. The so called glutamine addiction is a well-known effect observed when conducting cell culture and illustrates quite clearly the dependency that tumour cells exhibit on this amino acid. It is now known that glutamine breakdown provides by-products such as amino-acid precursors that are required by rapidly proliferating cells. Therefore glutamine has an anaplerotic role as the carbon source for the synthesis of α-ketoglutarate, an intermediate of the Krebs cycle. Furthermore, glutaminolysis in cancer cells highlights a connection between cytoplasmic and mitochondrial metabolisms, an issue that will be mentioned in this review because until recently it divided opinions. In this context, generalizations such as ‘tumour cells are highly glycolytic’ (1) or ‘glutamine metabolism is primarily directed at anabolic processes’ (2) should be taken with caution because they are true only in specific situations and experimental models. For example, taking the first statement one should note that tumour cells express the glycolytic phenotype only in specific microenvironments. In addition, many papers analysing the glycolytic flux do so by measuring the release of lactate. Frequently, authors fail to acknowledge the contribution of glutamine metabolism to lactate production and release. Regarding statement (2) it is important to keep in mind that in tumour cells, glutaminolysis contributes to lipid synthesis via the IDH (isocitrate dehydrogenase) pathway, and to maintenance of the redox equilibrium and ATP synthesis. Incidentally, IDH mutations have been implicated in a considerable proportion of gliomas and glioblastomas and myeloid leukaemia [1].

Many supporters of the classic Warburg effect sustained that glycolysis was sufficient for tumour cell survival and maintained that in these cells, mitochondria were actually dysfunctional [2]. Others were able to show that far from being dysfunctional, mitochondria from tumour cells were an integral part of the biochemical toolkit that allowed them to carry on dividing and successfully competing with the normal cells. Thus, it has become possible to envisage the tumour cell as highly adaptable units that are able to connect different pathways in order to overcome challenges that range from unfavourable environments to resistance to regulatory events such as apoptosis and anoikis. Nowadays there is a growing body of evidence to show that a tumour is composed of different cell populations that display different metabolic phenotypes. The different phenotypes represent adaptations imposed by the anatomical location within the tumour. Accordingly, if cells are situated near blood vessels where they have access to oxygen and nutrients, they may obtain energy from glycolysis and oxidative phosphorylation, whereas those located furthest away within the tumour mass resort to aerobic glycolysis as dictated by the prevalent hypoxic environment. The idea that cancer metabolism may involve more than just aerobic glycolysis has been reinforced by many recent studies on the metabolism of tumour cells [3]. Besides the more outstanding metabolic features that characterize tumour cells, other upstream elements are also important when describing the mechanisms of cell transformation. These include the genetic background, changes in oncogenes and tumour suppressors and as mentioned before the cell location in tumour mass. Furthermore, the peculiarities of the different models used for *in vitro* studies should be analysed carefully considered. Indeed, results obtained with cells in culture (with unrestricted access to oxygen), or those that use transfected constructs that transform cells through the overexpression of oncogenes should always be interpreted with caution. At any rate the recent papers have revealed and confirmed that cancer metabolism is more complex than originally thought and that tumours display a rather large collection of metabolic profiles [4]. This review will focus on central questions around the metabolism of tumour cells. For example, the unique mechanisms that are involved in the regulation of enhanced glycolysis and glutaminolysis and how do glycolysis, glutaminolysis and mitochondrial respiration liaise. Whenever applicable the differences between normal cells and tumour cells will be highlighted in order to show potential strategies for cancer treatment.

## GLYCOLYSIS

**Figure 1:**
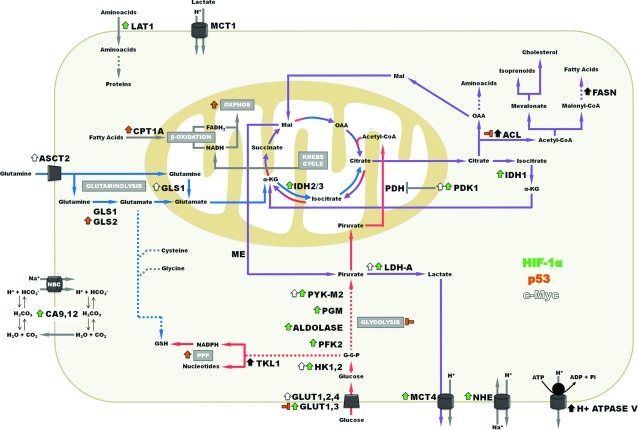
Energy metabolism in tumour cells The regulation by HIF-1α, c-*Myc* and p53 initiate the alterations in some metabolic pathways. Arrows represent alterations caused by the activation of HIF-1α (green arrows), p53 (orange arrows) and c-*Myc* regulation (white arrows) or others factors (black arrows). ACL, ATP citrate lyase; ASCT, neutral amino acid transporter; CA, carbonic anhydrase; CPT, carnitine palmitoyltransferase; FASN, fatty acid synthase; GLS, glutaminase; GLUT, glucose transporter; GSH, reduced glutathione; HK, hexokinase; IDH, isocitrate dehydrogenase; LAT, L-type amino acid transporter; LDH-A, lactate dehydrogenase isoform A, Mal, malate; MCT, monocarboxylate transporter; ME, malic enzyme; NBC, sodium bicarbonate transporter; NHE, sodium hydrogen exchanger; OAA, oxaloacetato; OXPHOS, oxidative phosphorylation; PDH, pyruvate dehydrogenase; PDK, pyruvate dehydrogenase kinase; PFK, phosphofructokinase; PGM, phosphoglycerate mutase; PPP, pentose phosphate pathway; PYK M2, pyruvate kinase isoform M2; TLK, transketolase; α-KG, alpha ketoglutarate.

There is general agreement that tumour cells display enhanced glycolysis [5]. The regulation of glycolysis can occur at various levels. For example, one could envisage different factors affecting the expression of each and every component of the pathway, beginning at the glucose transporters that promote the entry of the sugars into the cells. Besides regulation by transcription factors, modulation can be achieved by compounds formed along the sequential reactions of interconnecting pathways that accumulate and bind directly to the allosteric sites of the cognate enzymes. In the case of tumour cells, deviant metabolism could also be due to over–or underproduction of such intermediates. Metabolites that accumulate and promote progression to cancer have been coined as oncometabolites [6]. Thus, beginning with glucose transporters and proceeding along the pathway one could discriminate which members are up-regulated at the level of transcription. Hypoxia promotes the stabilization of HIF (hypoxia-inducible factor) 1 and 2, that in turn activate the expression of several other components of distinct signalling pathways. Incidentally, besides the glycolytic pathway, HIF-1α also extends its stimulatory effects to the TCA (tricarboxylic acid) cycle by activating pyruvate dehydrogenase kinase [7,8]. Within the context of the present discussion, the expression of all genes members of the glycolytic pathway, (except for phosphoglycerate mutase) including glucose transporters 1-3 are up regulated as a consequence of HIF-1α stabilization. HIF-1α is not the only transcription factor involved in up-regulation [9]. The c*-myc* gene which is overexpressed in a wide variety of cancers, normally responds to stimulation by growth factors. For example, in many tumours c*-myc* becomes constitutively expressed and is able to activate the expression, among others, of many of the genes coding for enzymes of the glycolytic pathway (Figure 1) [10]. Hence, it is not surprising that transformed cells display intense glycolysis as a result of the concerted reinforcement of expression of its constituent enzymes. Adding to this, the overexpressed enzymes themselves are subject to selection and as a consequence only certain isoforms are majorly represented in tumour cells. HK (hexokinase) is an example. Fast growing cells express primarily the HK-II isoform. Presumably, this isoform was selected due to the fact that HK-II binds directly to mitochondria and is thus able to capture newly synthesized ATP originating from the ATP synthase system as a substrate [11]. Pyruvate kinase, the enzyme catalysing the conversion of phosphoenolpyruvate to pyruvate, with the generation of ATP is also affected. This enzyme has been considered an important regulator of the entire pathway since the reaction catalysed by it is practically irreversible. Furthermore, the enzyme sequentially occupies the last position of the pathway. By increasing or lowering its activity, more or less pyruvate is produced, respectively. In the first situation, pyruvate kinase promotes catabolism by providing ATP and the metabolite pyruvate that can generate lactate or link glycolysis to the TCA cycle. In the second case, glycolysis intermediates accumulate and promote anabolism, with the concomitant production of amino acids, nucleotides and lipids. Most tumour cells contain an isoform of pyruvate kinase, PyK M2 (pyruvate kinase isoform M2), which is able to switch from one situation to the other. PyK M2 occurs as a tetramer (highly active), or as a dimer (less active). When cells require energy, tetrameric PyK M2 is prevalent. In contrast, dimeric PyK M2 becomes active when cells enter a proliferative stage. Control of PyK M2 gene expression occurs at the alternative splice level and not surprisingly involves oncogenes and tumour suppressors [12]. As alluded above, if on one hand there is ample evidence showing that the up regulation of glycolytic enzymes is consensual, complementary data obtained from functional approaches, i.e. measuring the activities of the intermediates of glycolysis is still relatively scarce. Detailed metabolomics studies should fill this gap.

## GLUTAMINOLYSIS

Glucose and glutamine are the most important energetic substrate for the cells. Glutamine is the most abundant amino acid in the body, being used in several pathways, including those of the nucleotide synthesis, oxidation in the Krebs cycle to produce ATP, and as a lipogenic and gluconeogenic precursor [13]. GA (glutaminases) converts glutamine to glutamate, releasing the the γ-amino group, which will be used to synthetize nucleotides, hexosamines and asparagine. In humans, there are two types of GA: the *GLS* gene (isoforms KGA and GAC–K-type) and the *GLS2* gene (isoforms LGA and GAB–L-type) [14,15]. Only GAC is compartmentalized in the mitochondria, being, in this context, the most important GA isoenzyme in anaplerotic processes [16]. This isoform is highly expressed in many types of cancer cells. Whereas normal cells use glutamine for synthesis of amino acids from the amide and amino groups, and nitrogen for *de novo* nucleotide formation [1], cancer cells secrete a significant fraction of glutamine-derived carbon and nitrogen, evidencing an enhanced activity of this pathway [17]. In some conditions, the cancer cells use glutamine preferentially to increase the ATP production by oxidative phosphorylation, in comparison with the biosynthetic production of the amino and amido groups.

The enhanced proliferation of cancer cells depends on a constant supply of nutrients among which glutamine is required to provide the building blocks for macromolecules, such as nucleic acid and proteins. This is carried out by using the amino and amide groups and the carbons skeletons in biosynthetic pathways. Glutamine also has a role in maintaining a high production of ATP through the Krebs cycle [18]. Glutamine contributes to citrate and lipid synthesis through the reductive carboxylation of α-KG by IDH, the surplus citrate being exported of mitochondria for lipid synthesis [19,20]. Even though proliferating cells are frequently found under hypoxic conditions, this does not attenuate glutamine catabolism. There are many instances in which cancer cells cannot oxidize normally the Krebs Cycle substrates and thus redirect the cycle through the reductive pathway. In this way, citrate is exported from mitochondria, and is used for lipid synthesis, maintaining a high consumption of glutamine maybe through the GAC isoform which is more expressed by the cells. This phenomenon was first observed in normal brown fat cells actively synthetizing lipids. Hypoxic cancer cells were found to synthesize glutamine derived lipids in the same way [21]. The glutaminolytic phenotype under hypoxic conditions, has been associated with activation of the oncogenes *Ras* and *Myc*, and the concomitant loss of function of tumour suppressor such as *p53* [22]. This increases the activity of the glutaminolytic pathway, enhancing the ATP and lactate production in cancer cells [1]. The fraction of glutaminolysis-derived lactate can be gauged by measuring its production when cells are incubated in the presence or absence of glutamine. Results by our group have shown that melanoma cells incubated in a medium containing glucose, but not glutamine reduce lactate secretion by almost 50% (N.D. Amoedo, M.F. Rodrigues, A. Galina, A. Cruz, J. Viola, J. Valencia and F.D. Rumjanek, unpublished work). Interestingly, when these cells are incubated with glutamine alone, the reduction of lactate is much more pronounced, a result that highlights the central role of glucose in many pathways. *Gls* expression is regulated by oncongenic expression of c-*Myc*, promoting tumour development, and the *Gls2* expression is regulated by tumour suppressor p53 [23]. The regulation glycolysis and glutaminolysis by c-*myc* suggest the mechanism to through which lactate is produced in large amounts by cancer cells (Figure 1). Equally important, *p53* induces *GLS2* expression, enhances mitochondrial respiration, ATP and GSH (reduced glutathione) levels and controls the levels of ROS (reactive oxygen species) [24].

In brief glutamine exerts a major role in the cancer cells, by acting as a precursor for macromolecules and by maintaining the ATP supply through the Krebs cycle, mainly through GAC function activated by c-*Myc* and by increasing the proliferation by the inactivation of p53 which consequently attenuates GLS2 expression.

## MITOCHONDRIAL FUNCTION IN CANCER CELLS–DIFFERENT VIEWS

The several metabolic adaptations observable tumour cells reflect the multiple facets of energy metabolism imposed by the different environments to which these cells are exposed. This diversity has contributed to misrepresentations stemming from Warburg's original observations and interpretations. This may have been partly due to the fact that although tumours do display enhanced aerobic glycolysis, these experimental observations should be taken as relative parameters with regards to oxidative phosphorylation. However, it must be borne in mind that the speed of the glycolytic flux is in fact a true gain in evolutionary terms and does confer certain advantages to the transformed cells, even if at a first glance, the energy balance as measured by ATP yield may seem disadvantageous when comparing glycolysis to oxidative phosphorylation. Furthermore, as is known today, hypoxia is not the only stimulus to aerobic glycolysis and cancer cells are known to subsist on precursors other than glucose [25]. With regards to mitochondrial function, one must also take into account the necessity of evaluating among others, parameters such as the protonmotive force, respiratory rate, ATP turnover, and mitochondrial membrane potential changes in isolated mitochondria and/or intact cells [26]. These data were conspicuously missing in early reports that conveyed the idea of a dysfunctional organelle. Finally, experiments carried out with cybrids (transmitochondrial cybrids), that is, mitochondria carrying homoplasmic point mutations may have helped to sustain the belief that mitochondria of tumour cells are somehow inoperative. As the techniques to obtain transmitochondrial cybrids involve complex manipulations and also do not entirely eliminate nuclear DNA effects on mitochondrial function, interpretations derived from this experimental model are not straightforward [27].

Indeed, recent data on the energy metabolism of tumour cells regard mitochondria as functional organelles and active participants in specific stages of the malignant transformation [3]. The general trend is to consider aerobic glycolysis as resulting from a selective process in which rapid synthesis of ATP is accompanied by flexible regulatory features that can shift the metabolism in ways that favour either energy production or anabolic reactions [28]. Indeed, mitochondria seem have an active role in generating oxidative stress and by doing so, promote tumour progression and the metastatic potential of cancer cells [3]. The same mitochondrial generated oxidative stress has been implicated in the so called ‘Reverse Warburg effect’ in which metastatic cells induce H_2_O_2_ mediated oxidative stress in stromal cells thus forcing them to engage in aerobic glycolysis and to produce lactate and ketone bodies to fuel oxidative metabolism in the cancer cells [29]. That mitochondria are fully functional in cancer cells has been eloquently demonstrated recently by Vaupel and Mayer [30] who showed that in tumours the pacemaker for oxygen consumption is the availability of the gas and not the respiratory capacity. Many other experiments involving mitochondria biogenesis (mitochondrial networking) have also shown that protein mediated mitochondrial fusion or fission can be correlated to tumorigenesis [31].

Clearly the picture of mitochondrial function is still not complete. However, patterns are emerging that suggest that the organelle is an important adjuvant in the progression and maintenance of the malignant state. Many details are still missing such as what regulates the crosstalk between the cytoplasmic glycolysis and the intramitochondrial oxidative reactions. Some clues are beginning to appear, however. Recent results have suggested that TRAP-1 [TNF (tumour necrosis factor) receptor-associated protein 1], a protein of the chaperone family) normally acts as a tumour suppressor by dampening mitochondrial respiration [32]. In tumour cells TRAP-1 expression is reduced and triggers enhanced respiration in mouse and human cell lines with a concomitant suppression of aerobic glycolysis. Many more of such switch factors will undoubtedly be found in the future which will help to understand how exactly the various biochemical pathways interact with one another and how they could become amenable to subversion. If on the one hand complexity presents an almost intractable problem to oncologists, on the other hand it is the stuff that evolutionist's dreams are made of. Complexity itself is at the core of cancer formation.

## CANCER CELLS CAN BE PREFERENTIALLY TARGETED: A CASE STUDY

The scenario of highly heterogeneous phenotypes makes it complicated to identify individual targets for the development of new and specific therapies. Nevertheless, many papers have described metabolic inhibitors with promising effects either alone or in combination with other drugs, as repressors of tumour development (reviewed in [33]). Different approaches are also applicable. Studies of Whole-genome sequencing and metabolomics are now being used as a powerful diagnostic tool to discover energy biomarkers in different cancer cells. An example was the discovery of oncometabolite 2-hydroxyglutarate initially in human glioblastoma multiforme and later in leukaemia [34,35].

Ideally, chemotherapy based oncologic treatment would be specifically directed at cancer cells by targeting metabolic pathways exclusive to them and by activating signals leading to apoptosis together with inhibition of growth (blocking anabolic pathways). Unfortunately, however, current chemotherapeutic drugs cause cardiotoxicity, neuro and nephrotoxicity because of off-target effects that are not immediately related to proliferation and DNA damage. Tumour features other than rapid proliferation and the underlying signalling pathways are now being scrutinized for possible pharmaceutical targeting. Anti-angiogenic treatment and tyrosine kinase inhibitors are typical examples. Nonetheless, these modalities may also elicit severe side effects, or may have only limited clinical use, e.g. Avastin and Iressa [36].

One additional problem is that many types of cancer cells have developed resistance to multiple hydrophobic chemotherapeutic drugs by way of the ABC (ATP-binding-cassette) transporters (hydrophobic pumps in plasma membrane) conferring an MDR phenotype (multi drug resistance). An attractive novel target is the altered energy metabolism of tumour cells which is now generally regarded as a hallmark of tumorigenicity and/or tumour progression. Thus compounds which may act on the glycolytic enzymes or mimicking metabolites as a glycolytic analogue may be considered as promising chemotherapeutic agents. A compound that has these features is the alkylating agent 3BP (3-bromopyruvate) [37]. Another advantage is that 3BP is not a substrate for the efflux pumps involved in the PDR (pleiotropic drug resistance) network that confers resistance to many anticancer and antifungal drugs [38–40].

**Figure 2:**
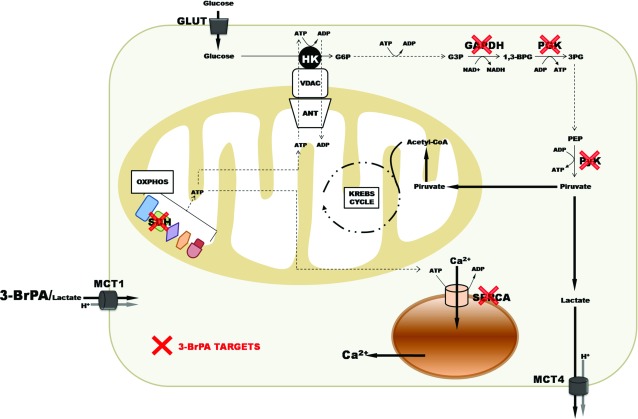
Main targets of 3BP in tumour cell 3BP is incorporated by MCT1 and reacts with this monocarboxylate transporter blocking lactate efflux ([44], see also the metabolomic data of this citation); (2) 3BP acts at distinct steps of ATP-producing enzymes, i.e. GA3PDH, 3PGK, PyK, malate, pyruvate, glutamate and succinate dehydrogenases (MDH, PDH, GDH, SDH, respectively), but does not affect the ATP-using enzymes, i.e. mt-HK, PGI, PFK I and SERCA [48]; (3) small metabolite analogues of the energy metabolism of tumour are not good substrates of efflux pumps involved in the chemotherapeutic resistance of several tumour cell lines. This peculiarity would facilitate the uptake and accumulation in cancer cells. The cellular redox system of reduced/oxidized glutathione levels [GSH]/[GSSG]_cyt/mito_ in cytoplasmic and mitochondrial compartments would play a role in 3BP mechanism of killing tumours cells. In addition to the amount of MCT 1, the amount of mt-HK binding to VDAC/ANT/F_o_F_1_ATP synthase would set the mitochondrial state more amenable to 3BP alkylation reaction.

Recently, mitochondria also became attractive targets because some enzymes and proteins which are crucial to the coupling between extramitochondrial and the intramitochondrial metabolism seem to be differentiated in tumour cells. HKs stand out due to their strategic localization on the mitochondrial membranes bound to VDAC and ANT. They face the cytosolic side of the outer mitochondrial membrane and help to maintain the membrane potential, regulate mitochondrial ROS production rate, block MPTP (mitochondrial permeability transition pore) formation, impair the release of cytochrome c from mitochondria and activate caspases during apoptosis. Thus, potential inhibitors of mt-HK (mitochondrially bound hexokinase) could have impact on the viability of tumour cells. The pioneering work of Pedersen's group [41,42] led to the discovery of the glycolytic analogue with high alkylating reactivity for -SH or -OH protein groups, 3BP that was shown to be a strong inhibitor of glycolysis. Pedersen's group identified mt-HK as the main target of 3BP [43]. However, recent observations by Pereira et al. [44] with human hepatoma cell line HepG2 showed that under more physiological conditions with 3BP at micromolar concentrations and measuring the recovered activities after the incubation of the cells with 3BP for 30-60 min, mt-HK, PGI and PFK1 (enzymes of the glycolytic block that uses ATP) were not affected by 3BP. The glycolytic enzymes that exhibited high reactivity to 3BP were GAPDH, 3PGK and PyK (Figure 2) [44]. The work of Pereira et al. also showed for the first time that 3BP inhibited the lactate release by the MCT 1 (monocarboxylate transporter 1) in HepG2 cells as analyzed by NMR using ^13^C glucose in intact cells. The importance of MCT 1 to cancer cells and the therapeutic use of 3BP was evaluated in a recent publication demonstrating that it participates in the entry of 3BP into the tumour cells [44,45]. Furthermore, the authors of this study [45] proposed that forced MCT1 expression in 3-BrPA-resistant cancer cells sensitizes tumour xenografts to 3-BrPA treatment *in vivo*. Thus, MCT 1 was proposed to be a potential biomarker for 3BP sensitivity and opens the possibility that the selectivity of cancer-expressed transporters can be exploited for the delivery of toxic molecules to tumours.

Given the importance of the carbons derived from glucose and glutamine for utilization and HIF expression for tumour cells, SDH (succinate desidrogenase) raises a promising target to 3BP inhibition. It was demonstrated that HepG2 pre-treated with only glutamine or with glucose plus glutamine as carbon sources affects the type of ETS (electron transport system) complexes inhibited by 3BP. With glutamine, using high resolution respirometry, it was possible to detect inhibition at the block of GDH/CI, SDH and decrease of ETS capacity. No changes were observed at the level of proton leak respiration that could be taken as evidence to show that under these conditions no MPTP are occurring in high degree. In contrast, with glucose plus glutamine as carbons sources inhibition of SDH only was observed with no decrease of ETS capacity. However, the results showed an increase in the proton leak respiration. This suggests that under these conditions MPTP may be occurring in a significantly high number of mitochondria thus triggering widespread apoptosis [44,46]. Using isolated preparations of mouse liver mitochondria, it was shown that besides SDH, other mitochondrial dehydrogenases may be alkylated by 3BP, such as SDH, PDH, MDH or GDH in response to respiratory state of mitochondria. The high rate of respiration coupled to ATP synthesis through ADP/ATP exchange mediated by ANT was positively correlated to the observed potency of 3BP inhibition. The inclusion of the GSH) promoted an almost total protection against the inhibitory effects of 3BP on mitochondrial respiration. Similar results were obtained using permeabilized HepG2 cells containing mt-HK able to support high ATP/ADP recycling rates confirmed that the respiratory ATP/ADP recycling turns mitochondria more sensitive to 3BP effects [47].

By assuming that most of the glucose entering cancer cells is phosphorylated at the surface of mitochondria (more than 80% HK is bound to mitochondria), the rate of glucose phosphorylation would tightly approach the equivalent rate of ADP used by the tumour mitochondria to synthesize ATP. Thus, we may assume an hypothesis: 3BP could kills the tumours cells by the following determinant factors: (1) increased amounts of MCT 1 may enhance the transport of the compound and produce lethal intracellular levels (Figure 2); (2) increased binding of mitochondrial HK binding to VDAC/ANT/F_o_F_1_ATP synthase would affect mitochondrial respiration and thus favour inhibition by 3BP; (3) by lowering the levels of GSH 3BP would not counteract the effects of ROS. Indeed it is known that 3BP is transported by MCT1 and that it acts simultaneously at distinct steps of ATP-producing enzymes, i.e. GA3PDH, 3PGK, PyK, MDH, PDH, GDH, SDH, but does not affect the enzymes that use ATP, i.e. mt-HK, PGI, PFK I and SERCA (sarcoplasmic/endoplasmic reticulum Ca^2+^-ATPase) [48]. Furthermore, small metabolite analogues of the energy metabolism of tumour such as 3BP are not good substrates for efflux pumps involved in the drug resistance of several tumour cell lines.

Like the 3BP example summarized above, many more drugs will undoubtedly be tested based on the data gathered on the metabolism of tumour cells. This is proving to be a very promising field of research. Drugs can now be designed to bind specifically to their targets so that in the near future as a result of the joint efforts of classical biochemistry, pharmacology and organic chemistry, it will be possible to rely on customized prescriptions applicable to all types of cancer.
